# A statistical optimization by response surface methodology for the enhanced production of riboflavin from *Lactobacillus plantarum*–HDS27: A strain isolated from bovine milk

**DOI:** 10.3389/fmicb.2022.982260

**Published:** 2022-08-25

**Authors:** M. Hemalatha, C. Subathra Devi

**Affiliations:** Department of Biotechnology, School of Bio Sciences and Technology, Vellore Institute of Technology, Vellore, Tamil Nadu, India

**Keywords:** *Lactobacillus plantarum*–HDS27, riboflavin, optimization, response surface methodology, central composite design (CCD)

## Abstract

In the present study, *Lactobacillus plantarum*-HDS27 strain isolated from bovine milk was used for the enhanced production of riboflavin. Production medium was optimized by one factor at a time with different parameters. Statistical optimization by Response surface methodology (RSM), central composite design was used to optimize variables such as pH, temperature, glucose, and yeast extract. The present study reveals the maximum riboflavin production by one factor at a time was obtained under the culture conditions; glucose, yeast extract, pH 6, the temperature at 40°C, and 3% of inoculum size. In RSM, analysis of variance for the responses was calculated. Among the tested variables, pH, yeast extract, and temperature showed significant impact on riboflavin production. Maximum amount of yeast extract in production medium resulted in increased riboflavin production. The riboflavin production after 24 h with the optimal condition was found to be 12.33 mg/L. It was found proximate to the expected value (12.29 mg/L) achieved by the RSM model. The yield of riboflavin was increased to 3.66-fold after 24 h with the optimized parameters. The current research, emphasizes that the *Lactobacillus plantarum*–HDS27 could be an excellent strain for the large-scale industrial production of riboflavin.

## Introduction

Riboflavin (vitamin B_2_) is an essential vitamin for the growth and reproduction of cells. Commonly, riboflavin is produced by both chemical and biological pathways (Massey, 2000). LAB is a group of industrially important microorganisms that are widely utilized in the food and dairy industries for the production of a variety of primary and secondary metabolites (LeBlanc et al., 2011). *L. plantarum* is one of the potential candidates for riboflavin production (Bhushan et al., 2020). Nowadays, the pharmaceutical industries are focusing more on probiotic bacterial strains producing vitamins (Singh Narayan et al., 2021). The product developed with probiotic strains producing vitamins will help in improving gut health and nutrition source for vitamin deficiency (Bhushan et al., 2021). In recent times, substantial developments are observed in the area of functional features of lactic acid bacteria specifically from starter cultures to microbial cell factories (Zhu et al., 2020). LAB are extensively explored for their ability to produce vitamin B group (LeBlanc et al., 2013; Thakur et al., 2020). LAB, also known as the dairy industry’s powerhouse and a source of health benefits as probiotics, have the ability to manufacture important biomolecules, particularly riboflavin. Given the widespread use of LAB in the food, pharmaceutical, and medical industries, as well as consumer demand for healthier meals, the utilization of these food-grade microbes as riboflavin manufacturers will be extremely beneficial to the forthcoming generation (Thakur and Tomar, 2015). For the industrial production of riboflavin, formulation of media ingredients is one of the important tasks. Optimization of process parameters is very important in enhancing the production rate of riboflavin.

In this current study, *Lactobacillus plantarum*–HDS27 was used for the riboflavin production and the process was optimized using a central composite design in response surface methodology (RSM). The production medium components glucose, yeast extract, pH, and temperature were optimized for over production of riboflavin. The primary goal of the current research was to enhance the riboflavin production and to design an optimized medium for over production of riboflavin by *Lactobacillus plantarum*-HDS27.

## Materials and methods

### Chemicals, growth media, and bacterial strain

Pure culture of *Lactobacillus plantarum*-HDS27 (Gen Bank accession number–MK314098) Production medium—De Man, Rogosa and Sharpe agar (MRS) and Chemically Defined Medium (CDM) (2.5 g lactose, 2.5 g K_2_HPO_4_, 3.0 g KH_2_PO_4_, 0.6 g ammonium citrate, 1.0 g sodium acetate, 0.25 g cysteine-HC1, 5.0 g salt-free, vitamin-free casein hydrolysate, 10 mL vitamin solution, 10 mL metal solution, 10 mL nucleic acid bases solution) were procured from Hi-Media Laboratories (Mumbai, India). Roseoflavin from Sigma Aldrich (St. Louis, MO, United States).

### Bacterial strain and inoculum preparation

During the course of optimization study *Lactobacillus plantarum*–HDS27, strain (Gen Bank accession number–MK31409) isolated from bovine milk was used. MRS medium was used for the maintenance of this strain. The pH of the medium was adjusted to 6.5. The organism was then inoculated on the sterile MRS medium. The growth rate of the organism was analyzed based on the OD value. When the OD value reaches 0.9–1.0, 1 mL of the culture broth was inoculated into 50 ml of seed medium and incubated at 37°C for 16 h on a rotary shaker at 120 rpm. After incubation, the cultured broth was centrifuged at 5000 *g* for 15 min. The pellet containing cell biomass was washed with 0.85% of saline. After washing, bacterial cells were suspended in 5 mL of sterile saline. From the suspension 1% inoculum was prepared for the production of riboflavin.

### Production of riboflavin from *Lactobacillus plantarum*–HDS27

The CDM and MRS were used for riboflavin production. The process was carried out by inoculating 1 mL of *L. plantarum*–HDS27 in both CDM and MRS medium. The inoculated medium was incubated in a rotary shaker for 48 h at 37°C. Growth of the strain *L. plantarum*–HDS27 was measured by observing the OD at 600 nm using a UV spectrophotometer and the riboflavin was estimated (Otto et al., 1983).

### Estimation of riboflavin

The amount of riboflavin produced was estimated after 48 h of fermentation. The fermented culture broth was centrifuged at 4800 *g* for 10 min. The collected supernatant was analyzed for the presence of riboflavin by using UV spectrophotometer at 444 nm. The concentration of riboflavin was calculated using extinction coefficient (1.04 × 10^–2^ M^–1^ cm^–1^) and the standard graph was plotted using riboflavin standard (Sigma Aldrich) (Sauer et al., 1996).

### Recovery of riboflavin

After 48 h of fermentation, the culture broth was checked for the pH (acidic) to dissolve the riboflavin. The pH was altered to 4.5 by adding concentrated HCl. Then the fermented broth was heated up to 121°C for 60 min to lyse the bacterial culture. To separate the lysed cells from the broth, the slurry was filtered by using whattman filter paper. The filtrate was centrifuged at 4800 rpm for 10 min. The supernatant was collected and pellet which contain remaining cell debris was discarded. To precipitate riboflavin, the collected supernatant was added with a reducing agent—sodium hydroxide and the pH of supernatant was adjusted to 9.0. Again to oxidize a reduced riboflavin, the broth was treated with high acidic solution—SnCl_2_ and the pH was reduced to 5.5. Finally, the precipitated riboflavin was separated from the fermented culture broth by vacuum drying (Kilbride et al., 1991).

### Extraction and purification of riboflavin

After drying, 5 mL of methanol was added to dissolve and collect the riboflavin from the dryer. Then, further, it was qualitatively analyzed by using Thin-layer chromatography (TLC) and High-Performance Liquid Chromatography (HPLC).

### Preparation of standard riboflavin

Standards of vitamins B_2_—riboflavin was purchased from Sigma (St Louis, MO, United States) and prepared at a concentration of 100 μg/mL (Kalingan and Krishnan, 1997).

### Qualitative analysis by thin layer chromatography

Thin-layer chromatography was done with the TLC silica gel 60 F_254_ aluminum sheet (Merck life science, Mumbai). Two different mobile phases were used. Butanol: benzene: acetic acid: water (8:7:5:3) and butanol: acetic acid: water (9:4:5) to separate riboflavin from the filtrated crude extract. Riboflavin was viewed under 254-nm ultraviolet (UV) light, or by fluorescence using excitation with 366-nm UV light. Retention factor (R_F_) values were calculated by using the formula **R_F_ = h_x_**/**h_0_** (Randerath, 1963).

### Analytical assay by high-performance liquid chromatography

For HPLC analysis, the separated yellow color compound in TLC sheet was scrapped using a sterile metal scrapper and diluted with methanol. Then it was further filtered by using a syringe filter with the pore size of 0.22 μm (Sigma Aldrich). According to Anyakora, the mobile phase, flow rate, and run time was done (Russell and Vanderslice, 1992).

### Optimization process parameters for enhanced production of riboflavin

To enhance the production of riboflavin in *L. plantarum*–HDS27, the production medium was optimized. In this study, five different parameters, carbon sources, nitrogen sources, temperature, pH, and inoculum size were selected for the optimization process. Glucose was used as a carbon source in production medium and it was substituted by different carbon sources (Glucose, galactose, maltose, sucrose, and lactose). Similarly for nitrogen source, instead of peptone, the medium was supplemented with yeast extract, ammonium chloride, tryptone, ammonium sulfate and glycine. The temperature 20–60°C was used for the optimization process. To find out the optimal pH for the riboflavin production, pH 3–7 was chosen. Inoculum size from 0.5 to 3% has been preferred for enhanced production of riboflavin. Riboflavin production and growth rate of *L. plantarum*–HDS27 were the two important parameters for optimization (Devi et al., 2015).

### Statistical optimization of riboflavin using response surface methodology

The production medium of riboflavin was optimized using a factorial central composite design with replicates at the central point and alpha points for four parameters. Glucose, yeast extract, temperature and pH are the four parameters. The lower factorial point, central point and upper factorial point were all designed as trial runs. The CCD studies featured a total of 30 experimental trials, including 16 factorial trails, 6 axial point trails, and 8 central point replication trails. The data were analyzed using the linear model. Two dependent variables were growth (response 1) and riboflavin production (response 2). The improved parameters that influence the responses were evaluated using 3D contour plots.

### Statistical analysis

The responses of experimental and predicted values were evaluated by means of analysis of variance (ANOVA). A second-order polynomial equation was used to assess the replies, and multiple regression analysis were used to fit the data to the equation. Later, a duplicate experiment was carried out to evaluate the predicted and observed values of the responses, using the optimum conditions for variables determined *via* response surface optimization. Design-Expert version 7.0 (Stat-Ease Inc., Minneapolis, MN, United States) statistical software was used to evaluate and interpret the outcomes of the experimental design (Yue et al., 2021).

## Results

### Production and estimation of riboflavin by fermentation

**FIGURE 1:**
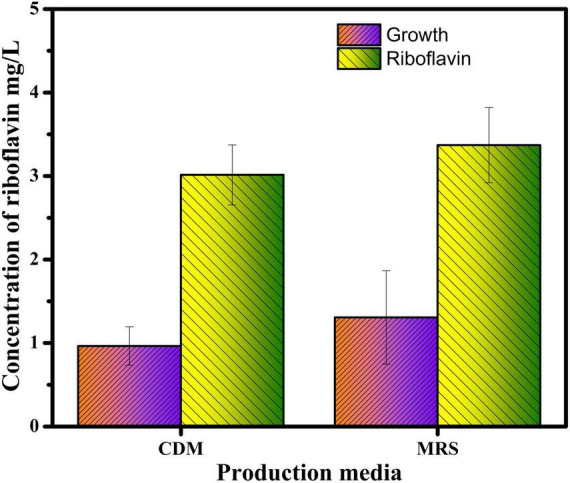
The growth and riboflavin concentration in CDM and MRS.

When compare to CDM, MRS medium showed better riboflavin production in *L. plantarum*–HDS27. On CDM, the production of riboflavin from *L. plantarum*–HDS27 was found to be 3.0 mg/L. The riboflavin production on MRS medium was found to be 3.37 mg/L. Experiments were done in triplicates and the average was calculated for the trials. The standard deviation (SD) was calculated and the error bars were used for the final calculation (Figure 1).

### Analytical assays

#### Thin-layer chromatography analysis

The RF value was 0.4 and it was found to be similar to the standard riboflavin. Comparatively benzene: butanol: acetic acid: water, were showing better resolution. Riboflavin spot was observed with fluorescence using excitation with 366-nm UV light. The above results showed that the strain *L. plantarum*–HDS27 actively produces riboflavin.

#### High-performance liquid chromatography

**FIGURE 2:**
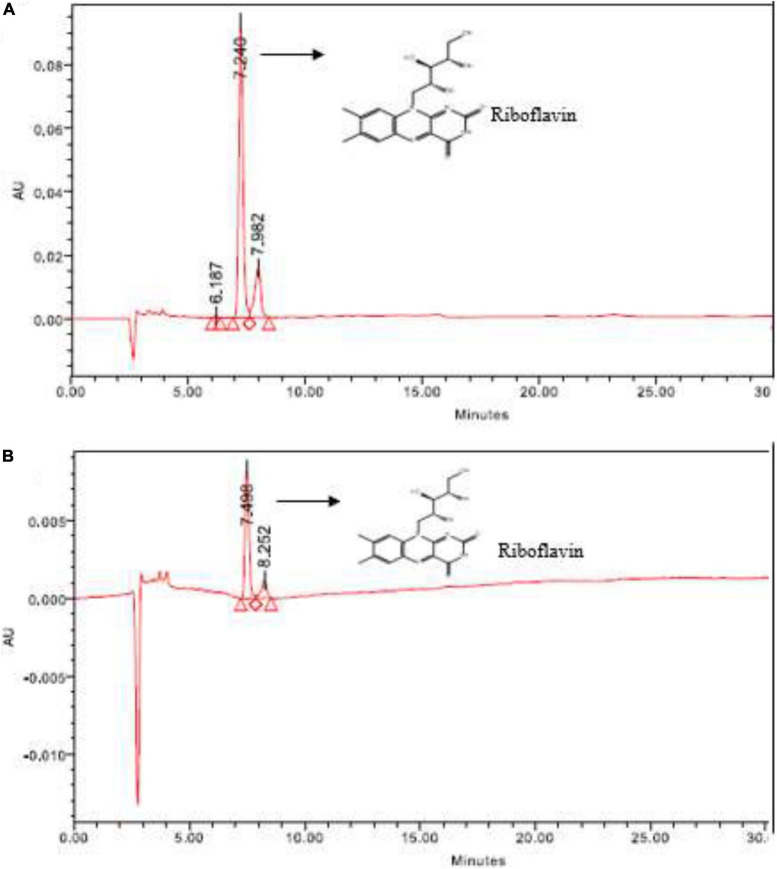
HPLC chromatogram of **(A)** standard—riboflavin and **(B)** sample—HDS27.

In HPLC, the target vitamin—riboflavin produced by *L. plantarum*- HDS27 was detected. The retention times (RT) of the standard and test samples were found to be 7.240 and 7.498. Both the RT values were closer and the sample was confirmed as riboflavin. Figure 2 represents the chromatogram of both standard and the test samples. The standard peak height was 93,254 and the corresponding concentration was 10 μg/mL. The test samples peak height was found at 8,325 and the concentration was found to be 3 μg/mL.

### Optimization

#### Carbon sources

**FIGURE 3:**
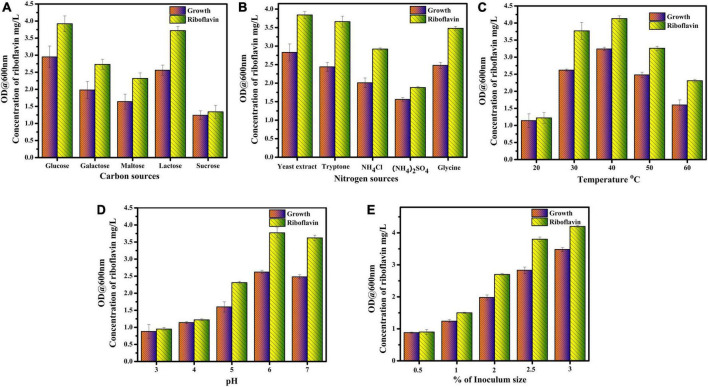
Optimization of riboflavin production with different parameters. **(A)** Carbon sources. **(B)** Nitrogen sources. **(C)** Temperature. **(D)** pH. **(E)** Inoculum size.

Comparatively, from the results, it was found that glucose was the best carbon source for riboflavin production. When the medium was supplemented with glucose as a carbon source, the production of riboflavin from *L. plantarum*–HDS27 was found to be 3.92 mg/L. The lactose was the second-best carbon source and the amount of riboflavin was found to be 3.72 mg/L from *L. plantarum*–HDS27. When galactose and maltose were used as a carbon source in the production medium, the concentration of 2.73 mg and 2.32 mg/L of riboflavin were obtained respectively. Medium supplemented with sucrose was showing less riboflavin production, than the other carbon sources and was found to be 1.34 mg/L (Figure 3A).

#### Nitrogen sources

The present study reported that yeast extract was found to be the best nitrogen source for riboflavin production. The *L. plantarum*–HDS27 produced 3.84 mg/L of riboflavin. The second top nitrogen source was tryptone and the riboflavin yield was found to be 3.66 mg/L. When the glycine was added as a nitrogen source in production medium 3.48 mg/L of riboflavin was obtained. The production medium supplemented with ammonium chloride produced moderate level of riboflavin from *L. plantarum*–HDS27 (2.92 mg/L). From the results, it was observed that the riboflavin production rate was decreased in medium supplemented with ammonium sulfate, it was found to be 1.88 mg/L (Figure 3B).

#### Temperature

The temperature plays a major role in growth and production of riboflavin. Commonly, the riboflavin production at 37°C was 3.37 mg/L. The optimum temperature for riboflavin production was 40°C and it was found to be 4.13 mg/L. The maximum riboflavin production was observed at 40°C. The strain *L. plantarum*–HDS27 exhibited good growth at 30 and 50°C and the amount of riboflavin obtained was found to be 3.77 and 3.62 mg/L. Growth rate and riboflavin yield was decreased to 2.31 mg/L when the temperature was increased to 60°C. The amount of riboflavin was reduced to 1.22 mg/L at 20°C. Figure 3C depicts the effect of temperature on growth and riboflavin production.

#### PH

PH is also one of the important parameter for the growth of *L. plantarum–*HDS27. From the results, it was evident that growth and riboflavin production rate was high at pH 6 and 7 and it was found to be 3.77 and 3.62 mg/L respectively. At very low pH, 3 and 4, the growth and production rate of riboflavin were reduced to 0.95 mg/L and 1.14 mg/L, respectively. A moderate level of riboflavin production (2.31 mg/L) was observed at pH 5. So, the optimum pH range for riboflavin production was found to be 6–7 (Figure 3D).

#### Inoculum size

When the percentage of inoculum increased, the concentration of riboflavin was also increased simultaneously. From the results, 3% of inoculum was found to be an optimum inoculum size and 4.2 mg/L of riboflavin was obtained. The growth and riboflavin production rate was reduced, when the medium was inoculated with 0.5 and 1% of inoculum and it was found to be 0.9 and 1.5 mg/L, respectively. When the percentage of inoculum was 2 and 2.5, the growth and riboflavin yield was moderate and it was found to be 2.7 and 3.8 mg/L (Figure 3E).

### Statistical optimization of riboflavin by response surface methodology

After 24 h of fermentation, *L. plantarum*-HDS27 produced 3.37 mg/L riboflavin in the pre-optimized MRS broth. Under ideal conditions (run 24), the same strain produced a maximum of 12.33 mg/L riboflavin. In the same experiment, the growth of *L. plantarum*-HDS27 strain OD at 600 nm was found to be 4.88. When compared to other models, the linear model was shown to be the “best fit model” for riboflavin production with the highest *F*-value. The regression model had very high significance when tested with an *F*-test with a very low error probability (P model > *F* = 0.0010).

By adjusting the parameters glucose (A), yeast extract (B), pH (C), and temperature (D) at various concentration (Table 1), the statistical design of the central composite model (CCD) was used to improve riboflavin synthesis (Table 2). Based on the findings, responses 1 (growth) and 2 (riboflavin mg/L) were achieved. Analysis of variance (ANOVA) was used to compare the reactions of predicted and experimental values to find if the polynomial expression could statistically predict the responses (Table 3). Then, CCD was used to optimize the factors with six central points. Response 1, the growth of *L. plantarum*-HDS27 was investigated, and the second-order polynomial equation was written as follows:


Response:1Growth(Y)1=+4.88+0.10*A+0.18*

⁢B-0.028*C+0.012*D+0.32*A*B+0.22*A*C

+0.13*A*D-0.065*B*C+0.039*B*D-0.074*C

*D-0.47*A⁢2-0.51*B⁢2-0.51*C⁢2-0.55*D⁢2

**TABLE 1:** Levels of independent variables in RSM for riboflavin production.

Variables	Factors	Lower factorial point (−1)	Central point (0)	Upper factorial point (+1)
A	Glucose (g/L)	10	15	20
B	Yeast extract (g/L)	10	15	20
C	pH	5	6	7
D	Temperature (°C)	30	40	50

**TABLE 2:** Central composite design and responses for optimization of riboflavin production medium.

Experimental trails	Factor A (Glucose g/L)	Factor B (Yeast extract g/L)	Factor C (pH)	Factor D (Temperature °C)	Growth of LP-HDS27 (Å600)	Riboflavin production (mg/L)
1	15	5	6	40	3.78	11.46
2	15	15	6	60	3.56	11.53
3	5	15	6	40	3.89	11.89
4	15	15	4	40	3.77	11.78
5	20	20	5	30	2.96	9.85
6	20	10	7	50	2.53	8.46
7	20	10	7	30	2.23	8.33
8	10	10	5	50	2.75	9.78
9	15	15	6	40	4.89	12.33
10	10	10	5	30	2.53	7.96
11	15	15	6	40	4.89	12.3
12	10	20	7	50	1.8	6.56
13	15	15	8	40	3.65	11.46
14	20	10	5	30	1.56	6.58
15	15	15	6	40	4.9	12.21
16	15	15	6	40	4.93	12.32
17	20	10	5	50	1.49	6.89
18	10	20	5	50	2.42	7.89
19	10	20	5	30	2.33	7.64
20	20	20	5	50	3.4	10.56
21	10	10	7	50	1.63	6.45
22	10	20	7	30	2.35	8.23
23	10	10	7	30	2.25	7.56
24	15	10	6	40	4.88	12.33
25	15	15	6	40	4.76	12.29
26	15	15	6	20	3.55	11.35
27	20	20	7	30	2.93	6.12
28	15	25	6	40	3.64	11.25
29	25	15	6	40	3.89	11.89
30	20	20	7	50	3.35	10.48

**TABLE 3:** ANOVA for response surface linear model [Response 1 (Y_1_) − Growth of *Lactobacillus plantarum* − HDS27].

Source	Sum of squares	df	Mean square	*F* value	*P*-value Prob > F
Model	6.33	10	0.63	2.88	<0.0010 Significant
A-Glucose	0.017	1	0.017	0.077	<0.0194
B-Yeast extract	0.59	1	0.59	2.66	0.7838
C-PH	1.57	1	1.57	7.15	<0.0150
D-Temperature	0.26	1	0.26	1.18	0.2904
AB	0.30	1	0.30	1.35	0.2599
AC	0.031	1	0.031	0.14	0.7133
AD	0.084	1	0.084	0.38	0.5439
BC	1.00	1	1.00	4.54	<0.0464
BD	0.18	1	0.18	0.82	0.3764
CD	2.30	1	2.30	10.42	<0.0044
Residual	4.18	19	0.22		
Lack of fit	3.41	12	0.28	2.56	0.5193 not significant
Pure error	0.78	7	0.11		
Cor total	10.51	29			

**FIGURE 4:**
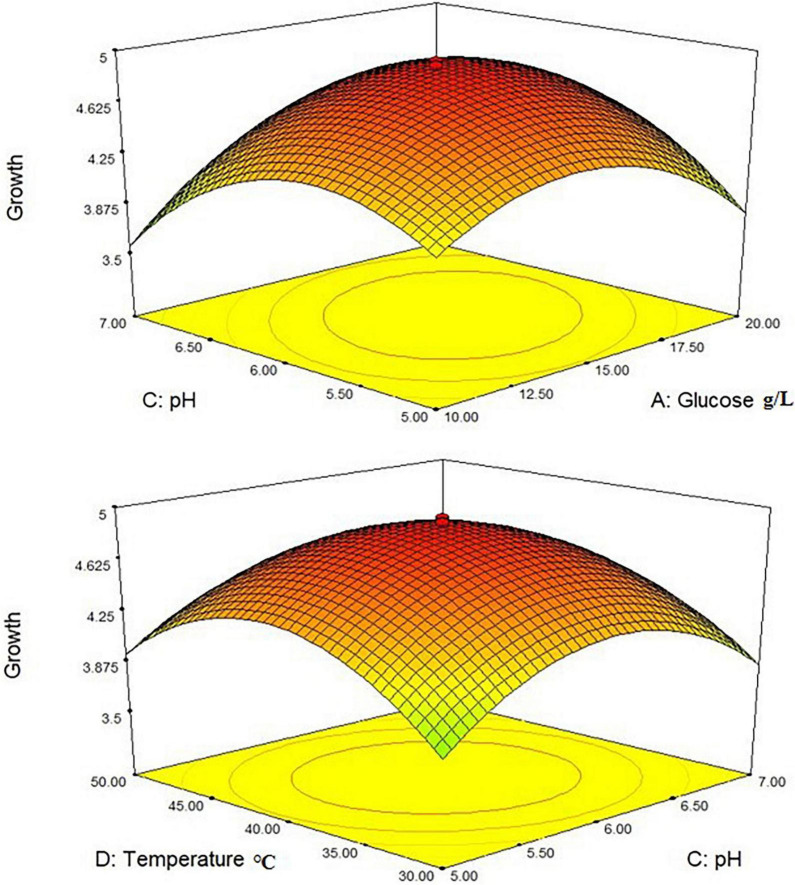
3-D interactions between the different factors of the medium optimized to increase the growth of *Lactobacillus plantarum* HDS27.

Where Y_1_ denoted the first response—*L. plantarum*-HDS27 growth and A, B, C, and D denoted the coded terms for the four test variables–glucose, yeast extract, pH, and temperature, respectively. Figure 4 shows the three-dimensional (3D) plots for the ideal levels of each variable for optimal riboflavin production. The 3D plots indicated a considerable influence on *L. plantarum*-HDS27 development either separately or in combination. Factor B, C, BC, and CD were found to be significant model terms. When compared to glucose (A) and pH (C), two processed variables, namely temperature (D) and pH (C) (Figure 4), had the greatest influence on the growth of *L. plantarum*–HDS27 when examined in linear terms. When these variables were analyzed in squared terms (*R*^2^), they all had a positive impact on growth (Table 3). The “Lack of Fit *F*-value” of 0.59 indicated that the lack of fit has not significant on the pure error. Non-significant–lack of fit was acceptable and the model was termed fit. The overall *R*-Squared value of the total determination coefficient was determined to be 0.9463, demonstrating a reasonable fit of the model to the experimental data. This also implies that 97% of response variance can be explained well, and that just 3% of the variability occur during the experiments. With a coefficient of variation of 3.13%, the adjusted determination coefficient value of 0.9610 proved that the model was highly significant. The value of 0.8758 for “Pred *R*-Squared” was in reasonable agreement with the value of 0.9720 for “Adj *R*-Squared.” The signal-to-noise ratio was measured using “Adeq Precision.” It was preferred to have a ratio of more than four. As a result, the 7.817 ratio indicated a sufficient signal, and this model can be used to navigate the design space.

Similarly, CCD with eight central points was used to optimize the factors, the response 2 of riboflavin production (g/L) was investigated, and the second-order polynomial equation was presented below:


Response:2Riboflavin(Y)2=+12.30+0.22*A+0.20

*B-0.23*C+0.21*D+0.51*A*B+0.25*A*C

+0.39*A*D-0.26*B*C+0.16*B*D-0.086*C

*D-0.68*A⁢2-0.82*B⁢2-0.75*C⁢2-0.80*D⁢2

**FIGURE 5:**
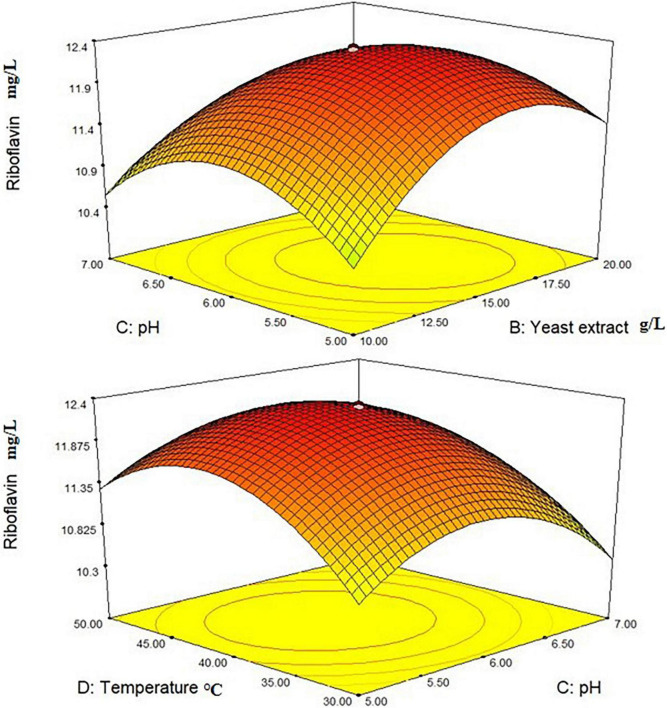
3-D interactions between the different factors of the medium optimized to increase the production of riboflavin.

Where A, B, C, and D were coded terms for the four test variables. Glucose, Yeast extract, pH, and temperature, respectively, and Y_2_ were the response 2 denoting the riboflavin production (mg/L). The 3D plots revealed that the *L. plantarum*-HDS27 had a considerable impact on riboflavin production (Figure 5). Values of Probability > F less than 0.1000 in response 2 suggested that the model terms were significant. B and C are determined to be significant model terms in this circumstance. When compared to temperature and glucose, yeast extract and pH had the greatest influence on riboflavin production when analyzed in linear terms.

The *F*-value for “Lack of Fit” is 0.64, indicating that the lack of fit has no effect on the pure error. Non-significant lack of fit is acceptable, and the model is referred to as fit. The determination coefficient was used to confirm the model’s quality of fit (*R*^2^). The value for riboflavin production in this investigation was 0.9085. The adjusted determination coefficient (Adj *R*^2^ = 0.8154) indicated that the model was highly significant. The higher correlation coefficient (r) value of 0.0976 indicated that the observed and expected values were well-correlated. The experiment’s reliability and precision were confirmed by a decreased coefficient of variation (CV = 14.44 percent). The “Pred R-Squared” value of 0.8857 was in reasonable agreement with the “Adj R-Squared” value of 0.8154. “Adeq Precision” is used to calculate the signal-to-noise ratio. As a result, the ratio of 18.638 indicated that the signal is appropriate, and this model can be used to explore the design space (Table 4). In response 2, the interactive effect of variables BC and CD were determined to be the most significant.

**TABLE 4:** ANOVA for response surface linear model [Response 2 (Y_2_) − Riboflavin mg/L].

Source	Sum of squares	df	Mean square	*F* value	*P*-value Prob > F
Model	62.86	10	6.29	2.08	<0.0108 Significant
A-Glucose	2.75	1	2.75	0.91	0.3513
B-Yeast extract	2.17	1	2.17	0.72	<0.0166
C-PH	8.80	1	8.80	2.92	<0.0138
D-Temperature	0.67	1	0.67	0.22	0.6428
AB	0.12	1	0.12	0.041	0.8413
AC	0.11	1	0.11	0036	0.8524
AD	0.13	1	0.13	0.044	0.8369
BC	8.81	1	8.81	2.92	<0.0038
BD	0.73	1	0.73	0.2	0.6291
CD	38.53	1	38.53	12.78	<0.0020
Residual	57.30	19	3.02		
Lack of fit	30.89	12	2.57	2.23	0.7356 not significant
Pure error	26.41	7	3.77		
Cor total	120.16	29			

The predicted and actual riboflavin production responses were very identical. The predicted values of riboflavin yield were estimated using ANOVA and compared to experimental data, demonstrating that the actual and predicted response values were quite close.

A statistical model was validated by using the RSM point prediction tool to find the best value for each of the four variables A, B, C, and D which were then utilized in the experiment. The actual riboflavin yield (12.33 mg/L) was close to the expected value (12.29 mg/L), supporting the model’s validity. Growth value was also significantly raised from 1.49 to 4.93 utilizing glucose (20 g/L) under optimal conditions. Several factors were used for improved riboflavin production by using a statistical technique. Comparatively, glucose and pH were found to be an efficient source for riboflavin production (12.33 mg/L) from *L. plantarum*–HDS27.

## Discussion

The current research focused on riboflavin production and optimization using *L. plantarum*–HDS27 a potent strain isolated from bovine milk sources. The present study reveals that the *L. plantarum*–HDS27 inoculated in MRS medium produced more riboflavin compared to the RAM (Riboflavin Assay Medium). According to Thakur and Tomar (2015) research reports, the strain KTLF1 (*L. fermentum*) and KTLP13 (*L. plantarum*) has been produced 2.13 mg/L, 2.36 mg/L of riboflavin in MRS and 2.71 mg/L, 2.54 mg/L in RAM (Riboflavin Assay Medium) (Thakur and Tomar, 2015). In the present study, the isolated strain *L. plantarum*–HDS27 produced 3.0 mg/L of riboflavin from RAM and 3.37 mg/L from MRS medium. Comparatively, the riboflavin production was 20% more than the previously reported strains. The strain isolated from fermented milk–*L. fermentum* MTCC 8711 produced 2.29 mg/L of riboflavin in chemically defined medium which was similar to RAM (Jayashree et al., 2010). In our study, the isolated potent strain *L. plantarum*-HDS27 showed 23% higher riboflavin production. According to Russo et al. (2014) roseoflavin resistant strain *L. fermentum* PBCC11.5 had shown 241 μg/L ± 38 of riboflavin. Comparatively, the isolated strain *L. plantarum*-HDS27 produced 3.37 mg/L of riboflavin, 14-fold higher yields than the other riboflavin producers. According to Burgess et al. (2006), *L. plantarum*, an overproducer strain isolated from milk showed 0.6 mg/L of riboflavin. In the current study, the same species *L. plantarum*, strain HDS27 isolated from milk samples collected from Vellore region showed maximum production of riboflavin (3.37 mg/L). According to Capozzi et al. (2011), two riboflavin overproducer strains of *L. plantarum* were used for the fortification in bread making process (Capozzi et al., 2011). The potent strain *L. plantarum*-HDS27 can also be used in industries for food fortification due to its maximum production of riboflavin. In another study, it was observed that the riboflavin production was enhanced by the inactivation of fol E gene in *L. fermentum* GKJFE, isolated from yogurt and the riboflavin production rate was 3.49 mg/L (Jayashree et al., 2010). Bhushan et al. (2021) reported different riboflavin producing strains of *Lactobacillus plantarum* BBC32B (319 ± 36 μg/L), BBC33 (304 ± 91 μg/L), BBC32A (276 ± 8 μg/L), and BIF43 (257 ± 91 μg/L) isolated from human feces and fermented milk. Except for some components, statistical examination of the data revealed that few interactions had a significant impact on riboflavin production and growth. Higher concentrations of glucose boosted riboflavin synthesis in *L. plantarum*–HDS27. It’s a good supplier of carbon sources. Similarly, Stahmann et al. (2000) reported a maximum of 15 g/L of riboflavin using glucose as a carbon source in *Eremothecium gossypii.* In this study, *L. plantarum*-HDS27 was not produced riboflavin in the early stages of fermentation using glucose as a carbon source. Riboflavin production was initiated after all or most of the carbon sources have been utilized by the organisms, similar findings were reported by Mickelson (1950) and Schlosser et al. (2001). Production rate of riboflavin was increased when the growth rate of the organisms decreased. The maximum riboflavin production was obtained at the stationary phase. Contradictorily, Wu et al. (2007) stated that K_2_HPO_4_ was one of the most important factors influencing *Bacillus subtilis* for riboflavin production. Based on the previous research findings, an optimal fermentation medium was designed using the ingredients (g/L): Glucose 40, ammonium citrate 2, K_2_HPO_4_- 10, yeast extract −10, Tween 20- 1, manganese sulfate- 0.1, sodium acetate 5, magnesium sulfate 0.4, at pH −6, and temperature 40°C. A maximum of 12.33 mg/L of riboflavin was obtained from the strain *L. plantarum* HDS27 grown in optimized medium. In comparison to the expected value of 12.29 mg/L, the validation run based on the optimum production in run 24 yielded the highest (12.33 mg/L) riboflavin output. The amount of riboflavin production was increased to four times when compared to the un-optimized medium (3.37 mg/L).

In the study conducted by Pujari and Chandra (2000), Plackett–Burman experimental designs were used to statistically optimize medium components for riboflavin production using *Erymothecium ashbyii*. In the improved medium, the yield of riboflavin was 1.134 mg/L. Comparatively, the isolate *L. plantarum*-HDS27 showed 10.8-folds higher riboflavin yield than the previously reported strain. According to Wu et al. (2007) optimum variables for riboflavin production using recombinant *Bacillus subtilis* RH44 have been determined by RSM based on a central composite design. The study reported, glucose, NaNO_3_, K_2_HPO_4_, ZnSO_4_, and MnCl_2_ were the optimal variables for riboflavin production. Similarly, the strain *L. plantarum* HDS27 also producing maximum amount of riboflavin in the medium enriched with glucose and K_2_HPO_4_. In another one study reported by Oraei et al. (2018), 13 minerals were examined for riboflavin optimization by *Bacillus subtilis subsp. subtilis* ATCC 6051, and fructose, MgSO_4_, K_2_HPO_4_, FeSO_4_, and yeast extract were found to be the best variables. The reported strain ATCC 6051 produced a total of 12.08 mg/L of riboflavin. The outcome was quite similar to the current investigation employing *L. plantarum*-HDS27 (12.33 mg/L) using yeast extract and K_2_HPO_4_. The statistical optimization of riboflavin production by *Rhodotorula glutinis* was done using the Box–Behnken design (Hassan et al., 2020). The riboflavin production was 88.25 μg/mL and the yield was 1.27 times higher in the optimized medium. In the current study, the isolate *L. plantarum*-HDS27 produced 12330 μg/mL of riboflavin which was 139.8 times greater than the previously reported strain *Rhodotorula glutins* (Hassan et al., 2020). Jayashree et al. (2010) has been employed *L. fermentum* for riboflavin production and the statistical optimization was done by RSM utilizing CCD. The best factors were peptone, beef extract, glucose, and K_2_HPO_4_, which produced 9.43 mg/L of riboflavin, which was four times greater than the basal medium. Similarly, *L. plantarum* HDS27 using glucose and yeast extract produced 12.33 mg/L of riboflavin, which is 1.3 times more than the previously mentioned strain, using the same experimental design RSM-CCD. So far, very few studies have been conducted in riboflavin optimization by statistical methods using lactic acid bacteria. This is the first report showing maximum riboflavin yield (12.33 mg/L) from the bovine isolate *L. plantarum* -HDS27.

## Conclusion

The main aim of the study was to identify the process parameters for the enhanced production of riboflavin from *Lactobacillus plantarum*-HDS27. The mass production of riboflavin using *L. plantarum* HDS27 strain in fermenter with optimized medium components will increase the yield of vitamin. The experimental value (12.33) was quite similar to the RSM model’s expected value (12.29 mg/L). Riboflavin is one of the most key vitamins used in day to day center and it is commonly produced in pharmaceutical industries. The strain *L. plantarum* HDS27 can be considered as a viable option for the food industry because it produces a maximum level of riboflavin in the optimized medium.

## Data availability statement

The original contributions presented in this study are included in the article/supplementary material, further inquiries can be directed to the corresponding author.

## Author contributions

CS conceived and designed the experiments and contributed to the protocol development as well as analysis. MH performed the experiments, abstracted the data, and prepared the manuscript. Both authors analyzed and interpreted the data, wrote and revised the manuscript, involved in data analysis, and approved the submitted version.
